# Understanding conversations about alcohol between parents and their 15–17 year olds: a qualitative study

**DOI:** 10.1186/s12889-018-5525-3

**Published:** 2018-05-16

**Authors:** Alexandra Sawyer, Lester Coleman, Richard Cooke, Lisa Hodgson, Nigel Sherriff

**Affiliations:** 1 School of Health Sciences, University of Brighton, Falmer, BN1 9PH UK; 2 Institute of Psychology, Health and Society, University of Liverpool, L69 3GL, Liverpool, UK

**Keywords:** Alcohol, Young people, Communication, Conversations, Qualitative, Parent

## Abstract

**Background:**

There is an increasing awareness that parents can play an important role in shaping their children’s attitudes towards alcohol and use of alcohol. However, there has been little research exploring the conversations parents have with their children about alcohol. The present study aims to address this gap by exploring conversations between parents and their 15–17 year old children.

**Methods:**

Using a cross-sectional qualitative design, recruitment took place over two phases to allow a purposive maximum variation sample of parents and young people. Sixty-four participants (*n* = 48 parents; *n* = 16 young people aged 15–17 years) took part in semi-structured interviews. The sample was diverse and included participants from throughout the United Kingdom. Thematic analysis was used to analyse the data separately for all parents and 16 matched parent-child pairs.

**Results:**

The parents’ findings were summarised within the following thematic areas: 1) style of conversation; 2) triggers to conversations; 3) topics conveyed during conversations; and 4) supervision of child’s alcohol consumption. Most parents were comfortable talking to their children about alcohol. It was considered that open and honest conversations helped demystify alcohol for young people. Most conversations that parents had with their children were brief and informal and a wide range of triggers to these conversations were reported. There was some indication that as children got older conversations became more frequent and more focused on safety. Overall, the matched parent-child interviews were very consistent regarding levels of child drinking, conversation starters, and topics discussed. However, in some cases parents underestimated their child’s need and desire for further conversations about alcohol.

**Conclusions:**

Most parents felt comfortable having conversations with their older children about alcohol. However, parents also wanted more support with having these conversations, particularly about how to start a conversation and what to talk about. This study provides several recommendations to help parents have an open conversation about alcohol with their children. For example, brief, informal chats seem to be the most appropriate way of speaking to children about alcohol compared to a more formal, “sit-down” style of conversation.

**Electronic supplementary material:**

The online version of this article (10.1186/s12889-018-5525-3) contains supplementary material, which is available to authorized users.

## Background

Young people in England typically first taste alcohol at age 13 and levels of consumption tend to rapidly increase as they advance in age [1]. Recent estimates show that 15% of 15 year olds in England had drunk alcohol in the last week, relative to 1% of 11 year olds [1]. Critically, the majority of 11–15 year olds who consumed alcohol in the last week drank above the pre-2016 daily recommended guidelines for adults, for each drinking day [2]. Further estimates show that by the age of 17, 64% of boys and 48% of girls drank on a weekly basis [3]. There is unequivocal evidence that frequent drinking and drunkenness is associated with adverse psychological, social, and physical health consequences [4]. In response to the concern of young people’s alcohol consumption, the first official governmental guidance on alcohol aimed specifically at children and young people was published in 2009 [5] stating that 15–17 year olds should only consume alcohol with the guidance of a parent and no more than once a week.

A range of factors impact alcohol use including genetic, family and peer influences, as well as wider social, environmental, and legislative contexts (e.g. [5, 6]). It is widely acknowledged that parents can play an important role in shaping their children’s attitudes to alcohol and use of alcohol [7, 8]. The idea that parents’ attitudes and behaviour can have a crucial impact on their children’s substance use is also in line with the assumptions of social learning theory [9], primary socialisation theory [10], and ecological systems theory [11]. Existing research on parental influence has tended to use quantitative methods to explore attributes of the family environment such as family structure as risk or protective factors for adolescent alcohol use. Furthermore, most studies have focused on adult “problem” drinkers, and their children, rather than “ordinary” families [8, 12]. In contrast, there has been less research on parent-child communication and how this may affect alcohol-related attitudes and behaviours [13]. A systematic review found evidence that parental modelling, limiting availability of alcohol to the child, parental monitoring, good parent-child relationship quality, parental involvement, and general communication (degree to which adolescents feel they can talk freely with their parents) were strategies associated with lower levels of later drinking by adolescents [14]. Highet [15] also found that communicating and negotiating with children seem to be effective strategies in helping young people develop and sustain a sensible relationship with alcohol.

There is a key gap in the evidence surrounding the conversations that parents have with older children about alcohol. A notable exception is a study conducted by Sherriff, Cox, Coleman, and Roker [16] which explored communication and supervision of alcohol in the family. This study showed that conversations between parent and child about alcohol were frequent, and that one of the main messages that parents tried to convey was that when used in a sensible and safe way, alcohol can be a normal part of adult life. However, many parents reported concerns, particularly how to open-up conversations around alcohol. Parents also expressed a strong desire for support in this area. The current study extends the work by Sherriff et al. by presenting contemporary evidence with a more geographically and demographically diverse sample (the sample in Sherriff et al., was mainly White-British parents residing in South-East England). This is particularly important given that recent evidence suggests that young people are engaging less in risky behaviours such as drinking, drug use, and smoking [17]. A report by the Institute of Alcohol Studies [18] found that although the proportion of underage drinking is still high there is evidence that this has declined. For example, in 2014 38% of 11–15 year olds in England had tried alcohol compared to 61% in 2003. A further limitation to the study by Sherriff et al. was the lack of a child’s perspective so it was not possible to examine the types of conversations that children find most helpful.

Therefore, the primary aim of this study was to explore conversations about alcohol between parents and older teenagers by conducting interviews with the parents of 15–17 year olds. A further aim was to explore young people’s views and compare their findings directly to their parents (as ‘matched-pairs’).

## Methods

### Design

The design was a cross-sectional qualitative study.

### Participants

Participants were parents and young people. In this report, “parents” refers to the primary caregivers of young children in the home. In addition to biological and adoptive parents, main caregivers may include kinship (e.g. grandparents), foster, and other types of caregivers. Eligible participants needed to: be a parent of child/ren aged between 15 and 17 years; provide informed consent, and; be able to understand and speak English. For young people to be eligible they needed to be able to give informed consent, with additional parental consent required to interview 15 year olds. Young people also needed to be able to understand and speak English.

### Procedure

During the recruitment period parent/s were invited to take part in a semi-structured individual interview regarding communication about alcohol between themselves and their children. The topic guide was designed to explore six key domains, partly based on Sherriff et al. [16] and discussions with the project steering group. These domains included: communication, supervision, modelling, legal and health issues, difficult issues, and mobile technology/social media (see Additional file 1). Children (15–17 year olds) of parents interviewed were also invited to take part in a semi-structured individual interview covering communication about alcohol between themselves and their parents (see Additional file 2).

All interviews were carried out over the telephone. Three of the authors conducted the interviews: AS (PhD, Research Fellow), LC (PhD, Research Fellow) and LH (BSc, Research Assistant), all of whom are trained in qualitative interview techniques. The interviewers would introduce themselves and explain the purpose of the research. No one was present apart from the interviewer. Interviews lasted approximately 30 min.

### Recruitment

The recruitment strategy took place over two phases which allowed a purposive maximum variation sample of parents and young people to be selected for in-depth interview (a method used to ensure a wide range of participants are included e.g. a range of sociodemographic backgrounds and differing levels of alcohol consumption).

For Phase 1, and to maximise the potential recruitment pool for both parents and young people, the research team engaged with an online survey panel who have access to 20 online panel providers. Panel partners randomly select respondents for surveys where respondents are highly likely to qualify. Each sample from the panel base is proportioned to the general population and then randomised before the survey is launched. An online screening questionnaire was launched to the survey panels. One hundred eligible participants completed the screening questionnaire and 40 agreed to further contact from the research team about taking part in the interview. The research team then contacted participants who expressed an interest to discuss the study in more depth, provided an information sheet about the study (usually via email), and arranged a date and time for the interview. At the end of the interview, parents were asked if the researcher could also speak to their child/children. If they responded positively, the child/children were provided with an age-appropriate information sheet, either via their parents or via email from a member of the research team. A member of the research team then contacted them directly to explain the study in more detail and arrange an interview. At the end of the interview parents were also asked if they knew of any other parents of children aged 15–17 who would be interested in participating. If no contact was made with participants after the first attempt, they were contacted a maximum of two further times (email, SMS, phone call).

Phase 2 of the recruitment employed additional purposive strategies to ensure underrepresented groups were included. Specifically, there was a need in this phase to recruit more parents of 16- and 17-year olds, single-parents, and young people. Further parents were invited through advertising in specific sites where these groups could be contacted (mainly leaflets and posters), such as local alcohol support services and university mailing lists.

### Sample characteristics

#### Parents

Of the 40 parents who agreed to be contacted from Phase 1 (online survey panel), 28 were interviewed (70% response rate) and three additional parents were interviewed after referrals from these parents. Seventeen parents were recruited and interviewed as part of Phase 2. Therefore, the total number of parents interviewed for this study was 48. Table 1 displays the demographic characteristics of these parents. The sample is diverse and includes participants from throughout the United Kingdom and from a range of ethnic backgrounds. Of the 48 parents interviewed, 38 (79%) had one child aged 15–17, and 10 (21%) parents had two children aged 15–17. Of these 58 children, 30 (52%) were boys and 28 (48%) were girls. 26 (45%) children were aged 15, 19 (33%) children were aged 16, and 13 (22%) children were aged 17. The average age of parents was 47 (ages ranged from 34 to 63). Based on drinking frequency and the amount and type of alcohol consumed parents were classified into the following five categories: abstinent (never drinks alcohol), very low (e.g. rarely, one or two drinks at special occasions), low (e.g. couple of drinks once or twice a week), moderate (e.g. one or two drinks three or four evenings week), and high (e.g. one or two drinks a night). Table 1 also displays the demographic details of the 16 parents whose children were also interviewed.

**Table 1:** Demographic characteristics of parents

	Total sample	Parents of children interviewed
	*N* = 48 (%)	*N* = 16 (%)
Gender		
Male	20 (42)	6 (38)
Female	28 (58)	10 (62)
Drinking level^a^		
Abstinent	6 (14)	0
Very low	9 (21)	4 (25)
Low	11 (26)	4 (23)
Moderate	12 (28)	6 (38)
High	5 (12)	2 (13)
Ethnicity		
White British	37 (77)	15 (94)
White American	1 (2)	0
White Italian	1 (2)	0
Black African	2 (4)	0
Pakistani	3 (6)	0
Other Asian	2 (4)	1 (6)
Mixed other	2 (4)	0
Religion		
None	26 (54)	10 (62)
Christianity	13 (27)	6 (38)
Catholicism	5 (10)	0
Islam	3 (6)	0
Buddhism	1 (2)	0
Marital status		
Married/Cohabiting	41 (85)	13 (81)
Partner (not living together)	1 (2)	0
Single	3 (6)	2 (13)
Separated/Divorced	3 (6)	1 (6)
Educational qualification		
None	1 (2)	0
GCSEs	5 (10)	1 (6)
A-Levels	18 (38)	5 (31)
Undergraduate	12 (25)	4 (25)
Postgraduate	11 (23)	5 (31)
Professional	1 (2)	1 (6)
Employment status		
Employed full time	22 (46)	5 (31)
Employed part time	13 (27)	6 (38)
Unemployed	6 (13)	1 (6)
Self-employed	5 (10)	4 (25)
Student	1 (2)	0
Retired	1 (2)	0

***Note.*** On occasions the percentages may not add up to 100% precisely due to the rounding up or down of decimal places. ^a^It was not possible to calculate drinking level for 5 participants because they did not state type/amount of drink, however, these 5 participants reported drinking 2–3 times a week

Postcode data were analysed using the indices of multiple deprivation (IMD) from the Office of National Statistics to gain an indication of the socioeconomic background of the interviewees. IMD scores are based on geographical location categorised by Super Output Areas and these range from 1 to 32,844 with a low score indicating most deprivation and a higher scoring indicating least deprivation. For the purpose of this research, IMD scores were categorised into quartiles to give an overview of the kinds of areas participants were drawn from. Analysis revealed that approximately 26% of the sample lived in the most deprived areas of England and 32% lived in the least deprived areas of England (see Table 2).

**Table 2:** Index of multiple deprivation (IMD) based on postcode data

IMD Quartile	*N* = 38 (%)
Most deprived	
Band 1 (1–8211)	10 (26)
Band 2 (8212–16,422)	2 (5)
Least deprived	
Band 3 (16423–24,633)	14 (37)
Band 4 (24634–32,844)	12 (32)

**Note.** One participant did not provide their postcode and this table only displays data for participants from England. Northern Ireland (*n* = 2), Wales (*n* = 5), Scotland (n = 2) all have separate indices. Of the five Welsh participants 3 participants were from the 50% least deprived Lower Super Output Areas and two participants were from the 30–50% most deprived Lower Super Output Areas. Of the two Scottish participants, one was from Band 1 of 5 (ranked 584 out of 6976 data zones, where 1 is the most deprived) and one was from Band 4 of 5 (ranked 5571 out of 6976 data zones). Of the two participants from Northern Ireland one participant was from an area ranked 316 out of 890 areas and another was from an area ranked 881 out of 890 areas (where 1 is the most deprived)

### Young people

Forty-seven parents were asked if a member of the research team could also speak with their child and 16 out of 47 parents agreed (34% response rate). Of these 16 children eight (50%) were boys and eight (50%) were girls; three (18%) children were aged 15, seven (44%) children were aged 16, and six (38%) children were aged 17. All young people interviewed were either in school or college.

### Data analysis

With participant’s permission all interviews were audio recorded, allocated a unique identifying number, and transcribed. Qualitative thematic analysis was used to analyse the data. Data analysis was guided both by the data collected and previous work in this area [16]. Braun and Clarke’s [19] phases of thematic analysis were followed. After transcripts were read and re-read to become familiar with the data, interviews were coded to generate an initial pool of codes. Codes were then collated into potential themes. Themes were reviewed by three authors (AS, LC, LH) in relation to the generated pool of codes and the entire data set. Finally, definitions and names were generated for each theme. Specialised qualitative data software (NVivo) was used to support this process. The analysis focused on: barriers and facilitators to conversations; comparisons in techniques to start conversations; perceived reactions and effectiveness of conversations; and suggestions for what would/would not ‘work’ through the parent-child interaction to reduce alcohol-related harm. Moreover, the sample was explored to see if findings could be analysed according to the variations within it including age of child, gender of parent/young person, drinking level of parents, and religion. Young people’s interviews were also examined alongside their parent’s interviews, which allowed exploration of how the nature and detail of the conversations concurred or differed between the children and their parents. Direct quotes are referred to by participant codes to ensure anonymity (P=Parent; YP=Young person; mother/father; participant number; age and gender of young person). Ethical approval from the University of Brighton was obtained.

## Results

This section presents the findings from all the parents interviewed (*n* = 48) followed by the findings from the parent and child pairs (*n* = 16).

### Parent findings

Findings from the interviews with parents are summarised within the following thematic areas: style of conversation, triggers to conversations, topics conveyed during conversations, and supervision of child’s alcohol consumption. The majority of parents were low to moderate drinkers and reports of drunkenness were rarely mentioned. Most parents reported drinking alcohol openly in front of their children. This was considered a positive way to show moderation and portray a sensible drinking message to their children. As might be expected a range of concerns were reported by parents regarding alcohol and older teenagers and these were primarily around safety and increased vulnerability. Specifically, parents were concerned about the immediate dangers to young people (e.g. crime, fights, being taken advantage of, risky behaviours) because of drinking too much alcohol and being out of control.

#### Style of conversation

This theme introduces the conversations that parents had with their children about alcohol including how parents feel about these conversations and the depth of these conversations. Overall, the majority of parents felt comfortable having conversations about alcohol with their children and found it easy to talk about. They thought it was important to have an open and honest dialogue with their children not only about alcohol, but all sensitive topics such as sex, drugs, and smoking. It was recognised that talking openly about these topics removed the taboo associated with them and if parents did not speak about them it would only create an air of intrigue around the topic:

*“I think if people don’t talk about it, it then becomes more of a thing to do.” (P9, Mother, 15-year old female)*

Interestingly, it seems that a few parents were motivated to do a better job than their own parents when it came to speaking to their children about alcohol (and other similar topics):

*“My parents…didn’t talk openly to me about things likes alcohol and drugs and it was always a bit of a taboo…in my twenties, I probably did make some mistakes in terms of my learning about sensible drinking and I do feel that my attitude is very much about giving kids information and not making these sort of subjects taboo, but talking openly about them.” (P14, Mother, 16-year old female)*

However, although parents thought it was important to talk about alcohol some parents reported not really having any specific conversations around alcohol with their child, primarily because their child showed no interest in alcohol and/or is perceived as being very sensible. For example, most of the 15-year olds in this study had only tasted alcohol in the home and, according to the parents, showed no interest in alcohol. Regarding the level of conversations that parents have had with their children, these were rarely big “sit-down” conversations but consisted mainly of brief, casual, regular talks over the years. It seems that parents view children as being most receptive to these types of conversations. As such, the majority of parents actively avoided formal sit-down conversations with their children about alcohol as they thought this style of conversation would be ineffective:

“*It’s important just to mention it whenever it comes up so that they feel comfortable. The moment you get a, ‘right, sit down kids, we’re going to have a talk’, they get that auto shut off thing where your parents are lecturing you so I don’t think they really take it in as much.” (P6, Mother of 15-year old female and 16-year old female)*

Finally, three adults in our sample were Muslim; a religion which prohibits alcohol. Importantly, even though all three Muslim parents reported not allowing their children to drink alcohol and that their children do not drink alcohol, they still thought it was important to have conversations with their children about alcohol because alcohol is prevalent in our society and they realise that their children may face pressure from their peers.

#### Triggers to conversations

Parents discussed a range of triggers and prompts that started a conversation around alcohol with their child but essentially it was very much about *“…taking opportunities to bring topics up* …” *(P31, Mother, 15-year old female).* These included when watching television together (e.g. the news, programmes where people are drinking alcohol/getting drunk, television adverts), social media, newspaper articles around alcohol, seeing people drunk, follow-on from lessons at school, and the first time their child tasted alcohol in the home. A few parents also noted that it was important to choose times to raise the topic when everyone was relaxed.

“*Some TV programmes like … where these people were getting so drunk they were ringing the emergency services all the time. You know, programmes like that, kind of brings it, what’s the word, that sometimes initiates the conversation.” (P9, Mother, 15-year old female)*

*“It’s best to be done at a point where there’s no pressure, and everybody involved is calm about it.” (P44, Father, 16-year old male)*

It seems that around age 16 children begin to experience alcohol for the first time outside of the home and for the young people in our sample this was primarily at parties. Parties were a common trigger for conversations around alcohol for this age group and often prompted the first significant conversation about alcohol:

“*It was just about a year ago when she was 16 and when she was going to her prom after party, or when they were talking about her prom and the after party and questions about where everyone is going and what alcohol they were going to bring. It was all very exciting about who is going to bring a beer and what not, so that was probably the first time.” (P41, Mother, 17-year old female)*

Some parents referred to family and friends who have problems with alcohol as a useful way of starting a conversation with their child about alcohol. It was thought that using a third person as an example helped raise the issue without appearing too direct or personal. The quote below shows an example where the problematic drinking of a parent was often a prompt for conversations with her daughter:

*“… we talk about her father’s [problematic] drinking, and that’s often a route in… because of his behaviour towards her and behaviour that she’s witnessed.” (P45, Mother, 16-year old female)*

Some parents also discussed that timing is crucial when speaking about alcohol with their child: *“it’s all about timing” (P9, Mother, 15-year old female).* Specifically, they did not think it would be useful to start a conversation *“out of the blue”* when there was no reason or relevant context (i.e. background of an actual incident). Some parents also highlighted the importance of listening to and actively involving children in any conversations about alcohol:

*“You’ve got to speak to them as you’re talking to them and then you want them to tell you how they feel. Yeah, it’s a two-way street, it’s a conversation; you listen to their point of view and maybe you know, it can work round whatever you need to work round.” (P9, Mother, 15-year old female)*

Although there were no obvious barriers to having conversations about alcohol with children, a few parents did mention that they sometimes struggled with finding the right balance between appearing too interested without invading the child’s privacy. Finally, children were also proactive in conversations with their parents and often came to their parents with questions prompted by similar things to what has been discussed previously (i.e. seeing someone drunk, someone at school who has got drunk, if they are out in a restaurant).

#### Topics conveyed during conversations

This theme describes the topics parents discussed around alcohol with their child. Parents reported discussing a range of generic short- and long-term effects associated with alcohol including hangovers, increased vulnerability, effect on behaviour, dangers of drinking alcohol, addiction, and impact on physical and mental health. Although parents thought it was important to discuss the negative sides of alcohol it was also important to convey that drinking can be a normal part of adult life if used sensibly and in moderation. The most common topics discussed related to sensible drinking and safety/harm-reduction strategies. Included in this were discussions around not drinking too much/avoiding binge drinking, being aware of their limits, strengths of different types of alcohol, alternating between alcoholic and non-alcoholic drinks, and the importance of not mixing drinks.

*“We told them there are different types of drinks, not only the brandy that we gave them to taste. And some people just drink beer, maybe on an occasion and they don’t get drunk but at the end of the day. It’s making sure that you can control what you are drinking and you don’t overdo it.” (P4, Mother, 16-year old male)*

Views on how to stay safe when drinking were also prevalent in parents’ accounts. Messages that parents tried to convey included: drinking water and eating before drinking, not leaving their drink unattended/drink spiking, keeping their mobile phone charged, calling parents if there are any problems, staying with reliable friends, and drinking in a safe environment. One parent noted that you may not always know what your child is doing and therefore it is important to provide them with information so that if they do drink, they do so sensibly and safely:

*“I’ve always said to make sure if you do have a drink and you go anywhere, just to make sure that they keep an eye on that drink and they have it with them all the time; they don’t drink something in a glass that somebody else has given to them that they don’t know what it is.” (P6, Mother, 15-year old female and 16-year old female)*

As might be expected, conversations about harm-reduction strategies and keeping safe were more prevalent with older teenagers.

*“He knows he gets a decent dinner before he’s allowed to go to the party because I always say ‘you need to line your stomach’. We always make sure that they’ve already booked their taxi to get home from the party, which are things we put in place to try and keep all the boys safe. Or we work out which parent is going to pick them up from the party. So we do put all of those in place and [son], before he goes out drinking, has always had to tell me where he is, which friend’s house, what’s the address, and what time he was leaving the party. And always has to text me when he’s leaving.” (P12, Mother, 17-year old male)*

Other topics that parents often spoke to their children about included drink-driving and peer pressure. There is some indication that peer pressure is more of a concern for the younger children in this study (15- and 16-year olds). Accordingly, parents of 15 and 16 year olds reported more frequent discussions about peer pressure with their children.

*“I don’t know whether it’s peer pressure. I think it was at the beginning when he was about 15, 16 and all the friends were starting to drink. I definitely felt it was then. Now, he’s happy to say ‘right, I’m not drinking at this party’ or ‘I am going to’. But definitely when he was 15 I would say it was peer pressure.” (P12, Mother, 17-year old male)*

One topic that was not so frequently spoken about with children was legal issues, perhaps because parents did not feel confident in their own knowledge to discuss this (topics that parents would like more support with are discussed below) or because it is a tricky area as most 17-year olds are already drinking alcohol. Interestingly, there was some discussion by parents regarding the *type of topics* that they thought were most effective in communicating about alcohol. Specifically, conversations were very much about the *“here and now”* as discussing long-term impacts may not mean very much to children. For example, it was thought that highlighting dangers to their children’s personal belongings would have more of an impact compared to discussing the long-term effects of drinking:

*“If I sat down and spoke to [son] about the damage he’s doing to his liver, I don’t think he’d even engage in the conversation. But if you talk about you could get phone stolen because you’re drunk, that would have more of an impact than the liver conversation.” (P12, Mother, 17-year old male)*

Although the majority of parents said they felt confident discussing most things to do with alcohol, some parents admitted that they lacked knowledge in certain areas. For example, some parents were not confident in their own knowledge to talk about units of alcohol, legal issues around alcohol, how to explain alcohol dependency to children, and types of alcoholic drinks that young people drink:

*“I suppose it’s my lack of knowledge about all the different types of alcohol. He’d mention a drink that I’d never heard of and therefore I’m immediately thinking well ‘what drink is that, is it strong’? So yeah, so my lack of knowledge is the modern alcohol of today that I’m not aware of. And so I struggle to have those conversations with him.” (P12, Mother, 17-year old male)*

It is also important to note that two mothers discussed difficulty talking to their children about the risks of girls being taken advantage of sexually when they had had too much to drink, despite thinking this was important to discuss with their children. Parents also recognised that conversations will change (including the topics discussed) with age and/or significant milestones such as learning to drive, drinking outside of the home and independent of parent-supervised parties, end of exams, and going to university. Within this it was recognised by some parents that once children are older (18+) and are drinking outside the home they will be reliant on *“whatever foundations they have built now” (P3, Father, 15-year-old twin males).* These findings suggest that conversations are rarely static within this age group (15–17 year olds) as they are faced with new perspectives, influences, and experiences on a routine basis.

*“I think there will be another conversation when he is 18 and can legally buy alcohol, that will be another one, probably on the birthday, ‘oh right you are ready to buy alcohol now and here are the rules’.”* (*P40, Mother, 17-year old male)*

Finally, many parents thought that there was not enough support to help parents talk to their children about alcohol. This is especially important in anticipation of future conversations with children as they get older. One mother thought there was less support generally for this age group (15–17 year olds) and another mother thought there was comparatively less support about alcohol compared to other topics:

*“… I see some literature or some things about danger, internet, and all internet, porn, drugs, …, sexually transmitted disease, but I don’t really hear about alcohol that much, for parents to talk about with teenager or children at all actually to be honest.” (P39, Mother, 15-year old male)*

Parents thought that resources would be helpful to increase their alcohol-related knowledge which would then help them speak confidently to their child about alcohol and answer any questions:

*“Somewhere you could actually go to if you do think that you need to sit down with your child and have a sort of open and frank discussion. Somewhere you can actually go that’s got all the answers that they’re going to throw at you and, yeah, a lot of useful information that you can give.” (P33, Father, 16-year old female)*

It was also thought that resources could be used to help initiate conversations with their children – “*a leaflet through the post is a reminder that you ought to be having these conversations*” (*P43, Father, 16-year old male*). Another parent suggested that within this general information there could be specific advice about how to start a conversation about alcohol, such as when watching the news and there is a feature about the dangers of alcohol. A range of formats for these resources was suggested by parents including websites, YouTube videos, SMS messages from organisations, and media (television and radio). A few parents also suggested that an online forum where they could go to for support would be helpful:

*“A forum where parents can sit down and help each other out because I’m sure parents all go through the same thing.” (P30, Mother, 17-year old male)*

There was also a preference for more traditional resources such as DVDs and leaflets, especially as there was some concern from a few parents over the authenticity of internet-based information:

*“So you don’t have to do much nowadays to Google something and find something on the internet, the issue is whether it’s a reputable source or not.” (P43, Father, 16-year old male)*

One mother said it is important to recognise that just providing information-based resources will not necessarily lead to parents talking about alcohol with their children. Rather, what is needed, are resources that are directed at breaking down the barriers to open communication that help adults talk to their children.

*“There may be some parents who want to have access to information but I suspect the reason that they [don’t] talk isn’t through lack of information but more because they are reluctant to talk.” (P16, Mother, 16-year old male)*

#### Supervision of child’s alcohol consumption

This theme describes the supervision strategies that parents have in place to manage alcohol and reduce alcohol-related harm in their children. The majority of parents did not ban their children from drinking alcohol outright. Most parents either allowed their child to taste alcohol, or allowed them to drink alcohol in the home and/or at parties. For many parents, this appeared to be a conscious strategy as they felt that banning alcohol would only serve to increase their children’s interest and make it more exciting:

*“If you take away that taboo, instead ‘okay, you can have one drink’ or … you know what I mean, it takes away that ‘oh I’m going to get absolutely blasted’ because … but there’s no fun in that because they’re allowed to have a bit of a drink.” (P9, Mother, 15-year old female)*

However, some parents have said to their children that they were not allowed to drink alcohol either because of religious reasons or because they thought it was better for their child to avoid alcohol. Many parents reported encouraging children to try their first taste of alcohol at home and/or to drink at home before they start drinking alcohol outside of the home. For example, some parents discussed a strategy of allowing children to taste alcohol to put them off it and essentially *“buy them a bit more time.”* Allowing a child to taste alcohol was also seen as a way of satisfying a child’s curiosity about alcohol and as a way to introduce a conversation about alcohol. Two parents described that they purposely bought strong flavoured alcohol for their child to taste so that they would be put off by the taste:

*“What my husband and I did, was … we brought brandy and beer and we gave him a little bit to taste and he was, when you looked at his face, you laughed, he was like ‘ooohhh’, it was burning his throat. We just wanted him … because he had started questioning, because his friends drink and they all do all sorts of things, and he wanted to know, so we just gave him so he would know, what’s it’s all about, yeah.” (P4, Mother, 16-year old male)*

Some parents also said that they allowed their child to have small amounts of alcohol in the home as they thought that this would be safer than drinking outside of the house. One father discussed that his daughter was going to her first party where there was going to be alcohol and he wanted to make sure she had a drink at home before so that she experienced its effects in a safe environment:

*“She’s going to a party on Saturday night, it’s the first party where the parents have said that alcohol’s acceptable. I’m sure they smuggled stuff in other parties, but it’s the first one that she’s going to, so I would rather she’d have had, drunk in our house and she can understand the implications of drinking before she goes and does it first thing at a party.” (P21, Father, 16-year old female)*

Specific safety and harm-reduction strategies they had in place were discussed by parents of the children who were already drinking outside the home (primarily at parties). Some of the strategies that were discussed included ensuring their mobile phone was charged, agreeing a time they had to be home by, texting parents when they arrive and throughout the night, and ensuring contact details of whose party it is has been provided. Of the parents who allowed their child to take alcohol to parties this was closely monitored with parents reporting that they would only buy drinks with low alcohol content (low strength beer or cider) and only allow them to take a few bottles. These examples describe clear safety strategies that parents have in place to reduce alcohol-related harm in their children. However, some parents suggested that there are more implicit strategies which are equally, if not more, important. Specifically, these parents have tried to convey to their children that they will always be there for the child no matter what. One mother described that as parents they can try and prevent their children from doing things, but that this is not always possible, so it is important that her child always knows that her parents will be there to support her:

*“I remember saying to her ‘we will always be here, we are here to catch you if you fall, be very very careful’ because ultimately we are just here to catch you.” (P11, Mother, 15-year old female)*

### Parent-child analysis

Young people’s interviews were explored alongside their parent’s interviews to see if and how the nature and detail of the conversations concurred or differed between the children and their parents. This was possible for 16 matched child-parent pairs. As this was a matched analysis of child-parent pairs, many of the results are reported on a case-by-case basis. The findings are presented below, within the following four thematic sections: 1) children and parents matching on levels of child drinking; 2) children and parents matching on conversation starters; 3) children and parents matching on topic areas; and 4) differences in children’s and parents’ accounts. Quotes are displayed in Table 3 and referred to in the text.

**Table 3:** Quotes for matched parent-child pairs

	Parent Quotes	Child Quotes
1. Children and parents matching on levels of child drinking		
a	*“On two occasions, there has been alcohol there [parties] and some of her friends had brought some alcohol. The last one she went to was New Year and at New Year she said she had some cider.” (Parent (P11, Mother) of YP10, 15-year old female)*	*“It’s only really been sort of twice when I’ve been at a friend’s party, so like when we were at New Year’s Eve, or just a friend’s birthday party. Yeah, I don’t really know, I just sort of had what was there. It wasn’t that much.” (YP10, 15-year old female)*
b	*“The week before when he went [to a party], we bought him a pack of, I think a box of 10, and he literally probably had one or two, even. And they were the small cans as well. He’s never been too fussed about it I don’t think.” (Parent (P47, Father) of YP9, 16-year old male)*	*“Yes, I’ve had it before but I don’t drink an awful lot.”* (*YP9, 16-year old male*)
c	*“When my ex-husband got married they let her have a glass of champagne, we do the odd thing like that, but generally she doesn’t like it.”* (*Parent (P41, Mother) of YP2, 17-year old female)*	*“I have once yes, but that was at a party but I haven’t touched it since…it was disgusting.” (YP2, 17-year old female)*
d	*“I think part of her thing is that she knows if she drinks too much she will be sick, she has a thing about vomiting, she has a fear of vomiting.”* (*Parent (P11, Mother) of YP10, 15-year old female)*	*“Yeah, I have a phobia of sick…The only thing that I remember is my mum talking about sick. Honestly, that’s all I remember. And saying that if I’m sick in her car she’ll never forgive me.” (YP10, 15-year old female)*
e	*“Certainly as far as my own son is concerned no…he’s got his head screwed on right with it. From what he says about some of the people who are at the parties with him, yes he comes back sometimes with ‘it was quite boring because everybody was paralytic and throwing up by 8.30 nine o’clock in the evening and it was dull’.*” (*Parent (P44, Father) of YP5, 16-year old male)*	*“Yes, definitely, when it gets to a point I think I should probably stop, I will stop and then I’ll drink something else like lemonade or water, for the rest of the evening or the time I am with my friends.” (YP5, 16-year old male)*
f	*“She comes back giggly from parties but she’s never been sick ... at the end of a party and I’ve collected her she’s always been compos mentis, you can always understand what she’s talking, she’s not slurring, she’s just a giggly, happy 17 year old.”* (*Parent (P46, Father) of YP4, 17-year old female)*	*“Mostly with friends, sort of, but not stupidly if that makes sense…I think I know it can be dangerous, but if you have it in moderation, and are sensible with it, it’s alright. So I think, I like to think I’ve got a responsible view on it.” (YP4, 17-year old female)*
g	*“I know from an early, a fairly earlyish age we let them have a small sip of whatever we’ve been doing. So they’ve tried beer, wine.” (Parent (P44, Father) of YP5, 16-year old male)*	*“My first proper alcoholic drink must have been a couple of years ago so I was 14…It was a small bottle of beer, when we were on holiday in France. Yes, it wasn’t particularly strong, my parents deliberately chose it.*” *(YP5, 16-year old male)*
h	*“So for instance now they’re in teenage age, they go to these parties, my daughter would take four bottles of Smirnoff Ice things.” (Parent (P46, Father) of YP4, 17-year old female)*	*“Yeah because even when we go to parties, we have to take our own alcohol, so it’s given to me, but it’s an amount that they’d be happy with me drinking…Yeah they’d only send me to a party with, they wouldn’t send me with too much alcohol, usually I have four drinks.” (YP4, 17-year old female)*
2. Children and parents matching on conversation starters		
a	*“No I think it was him, he asked me to purchase it because they are too young to buy it. So he has asked me to purchase it and I have willingly purchased it, because I would rather I go and buy this particular brand of lager or whatever rather than him trying to sneak off, and I don’t know just drink his friend’s vodka … it’s a small form of control I suppose.” (Parent (P40, Mother) of YP1, 17-year old male)*	*“I think it probably happened the night after I first had my first drink and then it just carries on after basically every time I go out, just a quick know your limits, beforehand.*” *(YP1, 17-year old male)*
b	*“Occasionally he would say like when he went to a party last week, we bought him some alcohol to take, but he didn’t drink much at all.” (Parent (P47, Father) of YP9, 16-year old male)*	*“Not an awful lot, just if I go to a party they will say don’t drink too much, be sensible, know your limits.” (YP9, 16-year old male)*
c	*“The first time we really discussed alcohol as a family we had gone to a family party and one of the relatives was really drunk and doing some very, saying some very silly things to them. And they were quite young at the time but they wanted to know what was going on.” (Parent (P35, Mother) of YP7, 15-year old male)*	*“One particular conversation was when I was a party and a family member got drunk, and they told us afterwards, basically not to get drunk.” (YP7, 15-year old male)*
3. Children and parents matching on topic areas		
*General points*		
a	*“Yeah we’re quite an open family ... we don’t talk a lot about it but it comes up round the dinner table conversations, when we’re in the car, walking the dog, it just comes up in general conversations, we don’t actually sit down with them and say ‘right you’ve got to listen to us’. We’re not that type of people, it just comes up in conversation.*” *(Parent (P46, Father) of YP4, 17-year old female)*	*“Usually we don’t really have sit down conversations and stuff, usually you’re out and about doing stuff or walking the dog.” (YP4, 17-year old female)*
b	*“I remember saying to her ‘we will always be here, we are here to catch you if you fall, be very very careful’, because ultimately we are just here to catch you, we can’t cover every single thing, we can’t achieve that, there’s only so much we can do, we have to prepare and keep her views and outlook as balanced as we can but at the same time she’s going to do something because kids do.” (Parent (P11, Mother) of YP10, 15-year old female)*.	*“I think it makes you more aware of alcohol and how to deal with it and I think they can often offer advice and like when they’re there and they’re not keeping anything over you and they’re letting you have your own freedom, but I think it’s important to let them know what goes on so that if you get into any situation they can help you out.” (YP10, 15-year old female)*
c	*“Kind of the safety aspects of it, I don’t think you can tell them you should never drink,* etc. *Because if you ban things they just want to do it more, so it should be how to moderate it, and how to do it safely. It’s no good saying, ‘oh you can’t drink until your 18 so we won’t bother telling you about it’. It’s, well if you do drink this is what can happen it’s the same sort of thing as with drugs if you take drugs this is what can happen. If you are drinking … please try and be safe with your friends and don’t overdo it.” (Parent (P40, Mother) of YP1, 17-year old male)*	*“I think it probably happened the night after I first had my first drink and then it just carries on after basically every time I go out, just a quick know your limits, beforehand.” (YP1, 17-year old male)*
d	*“Certainly with teenage girls spiking drinks and that sort of stuff, they’re aware of that, I think they’re told that. But the influences that being under alcohol, it makes you more happier so therefore you’re more flirty* etc.*, and people could take advantage of that.” (Parent (P46, Father) of YP4, 17-year old female)*	*“My dad did have a conversation with me the other week, we were on a dog walk, and talked about Uni and stuff, and he said, ‘you be careful about what other people can do to you when you’re drunk and stuff’. So obviously I know to be sensible with it, and people can put stuff in your drinks.” (YP4, 17-year old female)*
*Specific topic areas*		
e	*“We make a point about not mixing drinks or getting drunk in front of them or anything like that.” (Parent (P44, Father) of YP5, 16-year old male)*	*“Oh, talked about mixing alcohol, quite a few conversations about that and drinks that I should not mix because of the effects or if they are too strong.” (YP5, 16-year old male)*
f	*“He knows he gets a decent dinner before he’s allowed to go to the party because I always say ‘you need to line your stomach’.” (Parent (P12, Mother) of YP16, 17-year old male)*	*“Yeah they always said to eat before I go out.” (YP16, 17-year old male)*
g	*“I said to her ‘I think it’s quite important that you take a drink that hasn’t been opened’. I warned her about taking drinks where it’s been poured for her and she maybe doesn’t know who might have tampered with the drink and we had that discussion, and we talked about well, go and, see if you can find a drink that hasn’t been opened and then go and ask, see if you can find someone that can point you to where the bottle opener is or whatever.” (Parent (P14, Mother) of YP13, 16-year old female)*.	*“Yes, I keep my phone with me and I always take my drink with me wherever I go so I don’t leave it…Just about making sure that you open your own drink and making sure that you know it’s not been spiked, and not going over your limit.” (YP13, 16-year old female)*
4. Differences in children’s and parents’ accounts		
*Specific conversations not recalled*		
a	*Not recalled*	*“I’ve been told that it is better to have a* *** big meal*** * before you drink, and* *** drink water*** * in between.” (YP9, 16-year old male)*
b	*“She’d been very aware of the effects on some of her friends and she sort of described some of them, the way they were behaving, how some of her friends were becoming very emotional and tearful and one girl started opening up about her relationship with her father and things like that. So we discussed about the impact of alcohol on making people a bit* *** more disinhibited*** * and that sort of thing.”* (*Parent (P14, Mother) of YP13, 16-year old female)*	*Not recalled*
c	*“Well it’s not frequent it’s when he goes to a party or have a gathering with his friends which I don’t know, there has been one this year, one last month so I would say on average maybe* *** once a month*** *.” (Parent (P40, Mother) of YP1, 17-year old male)*	*“I drink when I go out with my friends…I’d say* *** every other week*** * maybe.” (YP1, 17-year old male)*
*Requests for more information from children*		
d	*“I suppose, well the* *** long-term effects*** * on your health of drinking too much and also the risks of becoming in a state where you’re not in control and so on. Those are the main concerns, I think that I would have.”* (*Parent (P42, Father) of YP3, 15-year old male)*	*“Probably slightly, I could choose to have that [more about alcohol] discussion, yeah…Probably the* *** effects that it has*** *, and maybe …,* ***maybe why people drink*** *…Yes because I suppose lots of people do it for lots of different reasons, so why they drink, why other people drink.” (YP3, 15-year old male)*
e	*“I warned her about taking drinks where it’s been poured for her and she maybe doesn’t know who might potentially have* *** tampered with the drink****…so we discussed about the impact of alcohol on making people* *** a bit more disinhibited*** * and that sort of thing…I put in place I suppose* *** some safety things*** * like any problems, she phone me straightaway, I can come and get you if …if something happens.”* (*Parent (P14, Mother) of YP13, 16-year old female)*	*“I guess [more information on] like the* *** dangers*** * because I’d take it more seriously if my parents told me…Yeah. Like the health effects and also like doing things that you will regret…I guess [more information] like* *** finding your own limit*** *, I am not really, I haven’t found mine yet.” (YP13, 16-year old female)*
f	*“I think, because I think he’s quite sensible around alcohol and* *** doesn’t feel that he needs me to talk about the warnings and the dangers*** * of alcohol because he’s not that keen on it.” (Parent (P12, Mother) of YP16, 17-year old male)*	*“Maybe the likes of the* *** differences between sort of like having some beers and then having sort of like vodka*** * and stuff. Because I think it’s a significant difference sort of … because if you have like a couple of beers it won’t have the same effect if you have sort of a bit of vodka.” (YP16, 17-year old male)*
g	*“Yeah, so I don’t have concerns about [daughter] at the moment, although I think, like all of us, we all need to be a bit concerned, because if you drink you’re at risk of falling over, apart from anything else, but whereas, my other daughter, I have much more concerns… So I’m lucky that my, that [daughter] is I would say quite responsible, as a person…Yeah* *** I would say their awareness is much higher than a lot of kids*** *, yeah.” (Parent (P45, Mother) of YP8, 16-year old female)*	*“Like really bad, I don’t really know all that much…Yes, it’s mainly things like, I think it’s obvious the higher percentage that is going to be more alcohol, than a lower percentage of things*. ***I don’t know what units mean****, I don’t know how much a unit is, or, because I know it says that women shouldn’t drink more than three units a day or something, but I don’t actually know how much that is. So things like that, that you should pick up when, as you get older.” (YP8, 16-year old female)*

#### Children and parents matching on levels of child drinking

The children in this study rarely drank (if at all), drank sensibly when they did, and did not report regular occurrences of drunkenness. In general, parents were aware of their children’s drinking experience and behaviours. Children and parents gave similar accounts of the amount of alcohol typically consumed, further illustrating the generally low level of consumption by the children in this sample (quotes 1a and 1b). Underpinning these low levels of drinking included negative attitudes to alcohol (e.g. not liking the taste) and a fear of vomiting, both of which were recalled by child and parent (quotes 1c and 1d). In all examples, the typical pattern was children drinking up to a certain point and then not continuing to drink to excess (quotes 1e and 1f). A particularly memorable occasion was parents introducing them to alcohol and this was the most frequent area of matching in terms of the child-parent accounts (e.g. quote 1g). This may be because this introduction was a deliberate strategy by parents, perhaps one that had been pre-planned which differs from the conversations about alcohol, typically “in passing”. For example, two parents had actively encouraged their children to try alcohol, some as young as 12 or 13 years old, in an attempt to normalise and demystify alcohol and ensure safety messages were in place prior to reaching the legal age. A further strategy recalled by children and parents, and in close relation to the encouragement to drink sensibly, were occasions where a sensible type or amount of alcohol had been supplied when going to parties (quote 1h).

#### Children and parents matching on conversation starters

The recollection of times where a conversation about alcohol was shared by both children and parents provides an indication of the most effective ways to start a dialogue. The purchasing of alcohol prior to a party was an opportune time to have a discussion and allowed parents to have some ‘control’ over ensuring the child would avoid coming to harm (quotes 2a and 2b). An additional account of starting a conversation about alcohol was where both the child and parent recalled a relative’s drinking (quote 2c). Again, perhaps different to brief conversations in passing, seeing real-life examples of drunkenness among family members was perceived to produce a more memorable experience, shared by both the children and parents.

#### Children and parents matching on topic areas

There was also matching on several topic areas and these can be considered as the most salient conversations that result in retained knowledge about alcohol among children. The findings are split into general points, followed by matched child-parent accounts of the more specific topics discussed.

##### General points

In general terms, children and parents shared accounts of the open nature of conversations “in passing” rather than a “big sit-down” approach, with the former universally agreed as the preferable option (quote 3a). As a further example of a general message being recollected, children were positive about their parents in helping them navigate their introduction to alcohol. For example, one young person noted the value of parental support and how this conveyed a right for independence and trust, whilst knowing that the parents were always available to help out if needed (quote 3b). This was a message that was also remembered by the parent. Many of the general topic conversations were in relation to safety and vulnerability (quotes 3c and 3d).

##### Specific topic areas

In addition to these general accounts, there were numerous examples of where the precise topics of the conversation were recalled. For example, there were matched conversations about not mixing drinks, dangers of drinking spirits, and eating before going to a party (quotes 3e and 3f). There was also one explicit illustration of where a topic of conversation raised by a parent, preventing drink spiking, was translated into actual behaviour by the child (e.g. Quote 3g).

#### Differences in children’s and parents’ accounts

The number of differences revealed between children and parents was less than the matched accounts shown above. To show the nature of the occasional differences, they are categorised as specific conversations that were not recalled and topics that children note they would like to know more information about.

##### Specific conversations not recalled

It was typical that not all topics were recalled by children compared to those raised by their parent. For all children where this occurred, this could be indicative of not remembering these conversations, not considering them as relevant, or just not referring to them in the interview (even though they may well have been remembered). A further angle on the occasional differences between children and parents was where the child mentioned issues that had been raised, but were not recalled in the parent interviews. Again, it is worth reiterating that whether this is a factual inconsistency (a truthful account of a mismatch) or a case of the parent not recalling a conversation that may have occurred, is questionable. For example, one young person talked about being told to eat a big meal and drink water – topics not raised by the parent (quote 4a). In another example, a conversation raised by a parent about the effects of alcohol and disinhibition is not mentioned in the child interview (quote 4b). A noticeable example of an inconsistent account was a parent who underestimated how often her son drank alcohol. The son drank alcohol twice as often as the parent believed he did (quote 4c).

##### Requests for more information from children

The second broad area of mismatch was concerning content that children wanted more information on, arguably a need that their parents were not aware of or were unable to address. There were a few accounts where children generally wanted to have more conversations with their parents about alcohol. Some children also wanted information on different topics than those discussed by their parents (quotes 4d and 4e). For example, one child wanted to know more about the negative health effects of alcohol and by contrast, their parent’s main concerns were around the dangers of mixing, the effects on disinhibition, and safety. In another example the difference in accounts is based on the parent’s assumption that her son does not want any more information because he sees it as not relevant to him. However, in the interview, the son identified a desire to know about the different strengths of alcohol and how to compare spirits and beer in terms of their effects (quote 4f). There was also an instance of a clear mismatch between what one child wanted to know and how this was not recognised or met by their parent (quote 4g). This child viewed her knowledge around alcohol as poor, but the parent perceived her knowledge to be higher than average. This mismatch is underpinned by the child and parent differing on the level of conversations about alcohol. The daughter perceived the information received as barely anything, whereas the parent recalled a conversation triggered by her father’s drinking.

## Discussion

This paper explored the conversations that parents have with their 15–17 year olds about alcohol. Most parents were comfortable talking to their child about alcohol. It was considered that open and honest conversations helped demystify alcohol consumption for young people. Most conversations that parents had with their children were brief and informal and a wide range of triggers to these conversations were reported. There was some indication that as children got older conversations became more frequent and more focused on safety. Overall, the matched parent-child interviews were very consistent regarding levels of child drinking, conversation starters, and topics discussed. Young people and parents generally reported similar accounts of their child’s alcohol consumption. The mutual recollection of conversation starters and topics provide some indication of the most salient and effective strategies to exchange knowledge about harm-reduction. There was also agreement regarding the style of conversation, which was viewed as an open and informal approach. It was fairly typical that not all topics were recalled by young people compared to those raised by their parents and vice-versa. Importantly, in some cases parents underestimated their child’s need and desire for further conversations about alcohol.

Conversations described in the current study share many similarities in terms of style, triggers, and content compared to previous research [16]. However, there were several interesting differences. Firstly, as might be expected ten years later, social media was discussed more by parents in the present study. For example, social media was used by some parents to start a conversation about alcohol, such as sharing a video on Facebook. Secondly, in the current study, most drinking occurred at parties rather than at public places or licenced premises, which could be because of stricter implementation of ID laws within the last decade (e.g. Challenge 25 launched in 2009 which encourages retailers to request identification from anyone who appears under the age of 25), which have made it harder for underage children to access and consume alcohol [18]. Consequently, parents reported more negotiations around providing alcohol for children to drink at these parties. Thirdly, parents in the current study highlighted the importance of conveying a message to their child that they can always call them if they need help without fear of being told off or judged. This was viewed just as important as the explicit safe and sensible drinking messages. Finally, there appeared to be a recognition that conversations will change (including the topics discussed) with age and/or significant milestones such as drinking outside of the home and independent of parent-supervised parties, end of exams, school proms (formal parties to celebrate an important date at school), and going to university.

Good communication with children has been associated with lower levels of adolescent alcohol-related harm [20]. Notably, the young people in this sample rarely drank and drank sensibly when they did, and did not report regular occurrences of drunkenness. Therefore, it is possible that the strategies that parents used to start conversations and the information that was portrayed can be considered as having a positive influence on young people’s knowledge, attitudes, and behaviour around alcohol. As such these techniques may be of great value to other parents. However, despite many parents feeling comfortable having conversations with their older children about alcohol many of the parents in our study thought that there was not enough support to help them talk to their children about alcohol. Therefore, in the same way that parents are offered support to speak about a range of sensitive matters with their teenagers (e.g. sexual health), it is important that support is also provided to parents to help them have conversations with their older children about alcohol.

This article provides a number of recommendations to help parents have an open conversation about alcohol with their children. For example, brief, informal chats seem to be the most appropriate way of speaking to children about alcohol compared to a more formal, “sit-down” style of conversation. Timing of when to start conversations is important. There needs to be some relevant context and it is best to choose a time when everyone is relaxed. Parents might want to use any relevant opportunities that occur, for example, when watching a programme on television. Although there were no major barriers reported that prevented parents from speaking to their child about alcohol, several topics were identified which parents might require more support with such as units of alcohol, legal issues around alcohol, how to explain alcohol dependency to children, and types of alcoholic drinks that young people drink.

### Strengths and limitations

This study provides an in-depth insight into the conversations that 48 parents have with their 15–17 year old children about alcohol. A unique aspect to this study is that 16 children of these parents were also interviewed, which allowed parent’s responses to be compared with their child’s. Another strength of the study was the diversity of the sample with participants recruited from across the UK, a gender balance with respect to young people and parents, and parents from a range of black and minority ethnic backgrounds. However, interpretation of the findings and recommendations should be made in the context of the following considerations. Firstly, with regards to the sample, single-parent families, parents with lower levels of education (i.e. no educational qualifications or GCSEs), young people who are heavier and more frequent drinkers, and parents who experience problems with alcohol are underrepresented in this study. Therefore, the current findings should not be generalised to non-traditional families, families who experience problems with alcohol, and parents with lower levels of education. Secondly, the parents and young people that opted to participate in a study about conversations may be those who are not having difficulty with conversations about alcohol. Therefore, there is a possibility that the sample in this study may represent a group that is more confident about communicating with one another than the general population. Thirdly, parents’ and young people’s self-reported alcohol use may also be different to what they are actually drinking (either via under- or over-reporting). However, the detail provided in the interviews would suggest this was not the case for most of the participants in this study.

### Future research

The findings suggest that conversations parents have with their 15–17 year old children are likely to change again when children are older (i.e. when children reach the legal age to drink alcohol, when they start drinking at pubs/clubs, and when they go to university). As far as we are aware there is little research which explores conversations parents have with older age groups (18+ years), therefore it would be of interest to explore these in more depth. In the current study conversations did not vary according to the gender of the young person or parent, or level of parent drinking. Future studies should explore further if and how conversations vary according to characteristics of parents and their children. For example, previous research suggests that parents use different communication strategies when speaking to boys or girls about alcohol use [21]. Although the focus of this study was on parental conversations about alcohol it is recognised that peer groups are also an important influence for this age group [22]. Therefore, future studies could further explore if and how peer groups influence parent-child communication about alcohol. Finally, although qualitative studies provide an in-depth insight to parents’ and children’s experiences of conversations, longitudinal studies are needed to extrapolate the effect of parental conversations on child alcohol use and alcohol-related harm.

## Conclusion

This study provides an in-depth insight into the conversations parents have with their older children about alcohol. Despite most parents feeling comfortable having conversations with their older children about alcohol many parents still felt that there was not enough support available to them regarding how to start these conversations and what to talk about. Therefore, it is important that more support is available to parents to have these conversations. The young people in this sample rarely drank and drank sensibly when they did. Therefore, this study tentatively suggests that conversations with older children about alcohol can have a positive impact on young people’s knowledge, attitudes, and behaviour around alcohol. The findings from this paper provide several recommendations to help parents have an open conversation about alcohol with their children. This study makes an important contribution to the evidence-base for resources to support parent-child conversations around alcohol, which can be furthered by future research among a sample where alcohol is more frequently used.

## Additional files

Additional file 1: Topic guide - parents (DOCX 27 kb)

Additional file 2: Topic guide – young people (DOCX 26 kb)
