# Supplementary material for: Understanding conversations about alcohol between parents and their 15–17 year olds: a qualitative study
Source: BMC Public Health. 2018 May 16;18:631. doi: 10.1186/s12889-018-5525-3 (PMC5956849; doi:10.1186/s12889-018-5525-3)
Supplement: Supplementary file 1 — Topic guide - parents (DOCX 27 kb) [file 12889_2018_5525_MOESM1_ESM.docx]

**TOPIC GUIDE – INTERVIEWS WITH PARENTS**

1. **Introduction**

Before we start properly, can I just ask a few questions about yourself and your family to provide a bit of context to the rest of our discussion? (read demographic questionnaire)

1. **Alcohol in the home – parent and child’s use** *(introduce the topic of alcohol use in general terms and explore parents attitudes including any principles they have around alcohol whether personal or institutional including religion)*

a) Do you drink alcohol at all?

- *If yes, ask how much, how often? Who with? In or outside the house? In front of children? How much do you think your children know about what you drink, how often and where – do they do this with you, see you etc.*
- *In general, do you think your drinking style has an effect on your children? Do you think they learn anything by looking at the way you drink?*
- *If no, do you think a parent’s drinking style may have an effect on young people?*
- *If no, – do you have any views about alcohol in the home, such as its visibility or consumption? This could be a personal viewpoint or an institutional one from religion or wider belief system.*

b) As far as you know, has your child ever drunk alcohol?

- *If yes,* *how has their drinking developed over the years (first drink, frequency of drinking, etc.)? Do you know roughly how much they drink (including getting drunk), what they drink, how often they drink, who they drink with and where they drink?*
- *If no, why do you think that is? e.g. not interested, it hasn’t been offered to them, they think it’s dangerous, they don’t like the taste or smell.*

1. **Worries about young people and drinking *(setting the context for talking about alcohol)***

a) Do you have any concerns about young people and drinking alcohol?

- *Health issues (being sick, falling over, getting into fights, damage to body, being alcohol-dependent, link to other health-damaging behaviours like smoking);*
- *Practical issues (where they drink, type of alcohol, availability to young people, amount they drink, costs);*
- *Regretted events including social media interaction, regretted sex, etc;*
- *Drinking culture;*
- *Missing school/college.*

1. **Talking to children about alcohol with view of importance and examples** ***(moving from alcohol use to talking to children about alcohol – most important question to explore in depth)***

In general, do you think children need to have information about alcohol? If so, how should it be provided and by whom?

*Do you think it is your role to talk about alcohol with your child? (compared to school, other sources, etc.)*

1. What do you think should be covered in the information provided to children about alcohol? (e.g. risks, benefits, ‘how to drink’)
2. Who do you think your child would talk to if they wanted more information about drinking and alcohol? *Probe. yourself/your partner, siblings/other relatives, friends, teachers, GP.*
3. In general, do you think it is important to talk about alcohol with your child?
4. Have you spoken to your child about alcohol? For example, about its effects, how to stay safe, legal issues?

- *Ask parents to expand on the details of the conversations that they have had with their child – to include how it was prompted and information/topics conveyed, including child reaction (explored in more detail in later questions)*
- *How easy was this conversation(s)?*
- *When did the first conversation you have occur? (separate out pre 15 and post 15- year old conversations)? For those with children over 17, what lessons have they learnt that are conveyed to 15-17 year olds? Any regrets (should have talked more when they were younger, etc.)*
- *Have you employed any strategies to deal with the worries reported earlier (Q3)?*
- *Have you used or referred to any social media (websites etc.) when talking to your children?*

1. If you haven’t spoken to your child about alcohol can you tell me why not?

- *Is it something you see as particularly important or not really?*
- *Have you wanted to speak to your child but couldn’t?*
- *Are you planning on speaking with your child sometime in the future?*
- *When do you think you will (e.g. suitable opportunity)?*

1. **Talking in more detail - Raising the issue *(moving into further detail about conversations starting with raising the issue)***

a) How did(do) the topic first come up? Did(do) you or your child raise the issue?

- *Were there any triggers for conversations (e.g. initial drunken episodes, school disco, birthday parties, break ups)?*
- *Easier to respond to their questions or easier to start the discussion with them?*
- *What do you think have been the most useful ways to start a conversation with your child? Appropriate language, resources, etc.*

b) When do you think are the best times to talk to your child about alcohol?

- *Opportune moments, at dinner, when something has triggered the need to talk about alcohol (personal, family, or peer experiences), child’s interest/curiosity*

1. **Talking in more detail – Topics *(moving from how to start conversations to information/topics shared)***

a) What types of things have you discussed with your child regarding alcohol?

- *Information about alcohol in general;*
- *Legal issues;*
- *Safety issues (Identify where discussion of* *alcohol-harm reduction occurs in these conversations);*
- *Rules/expectations;*
- *Peer pressure;*
- *Reasons for concern.*

b) Have you referred to social media in anyway when talking to your child about alcohol/helping you speak to your child about alcohol? E.g. websites, trending stories etc.

1. **Examples of conversations *(to focus on specific examples if this hasn’t arisen already in the interview to capture the precise details of what was said, when etc.)***

a) I’d like you to think about some instances where you’ve had a conversation about alcohol – think about one example where you felt it went well (like got some useful information across, raised at a good time etc.)

b) And now think about a situation where it didn’t go so well – why was that?

c) Have you ever had to manage a situation where, for example, your children wanted to drink alcohol in the home with friends, or bought alcohol for them?

d) Have these conversations you’ve had with your child about alcohol changed as they’ve got older (raising issues and information/topics) – think about when they were in their early teens (13-14) compared to now?

- *If you had conversations with your child when they were younger did the conversation differ to those you have now? If yes, in what way are they different?*

1. **Most/least effective conversations** and additional ways parents have influenced their children’s alcohol attitudes, information and use ***(strong focus on a particularly effective conversations and other strategies so that such real-life tips and techniques can be shared with other parents)***

a) Overall, what do you think have been the most effective conversations with your child about reducing current/future use or harm from alcohol?

b) What evidence do you have for this? Did you notice any change in their attitude or use of alcohol?

c) Have you talked to them about the best ways to reduce harm when drinking (and how)?

- *Eating properly;*
- *Plans to return home safely;*
- *Drinking in groups and ‘looking out for each other’;*
- *Watching their drinks (spiking etc.);*
- *Nominated lower level drinker;*
- *Social media to ‘check in’ with friends/family.*

d) Any examples of the least effective conversations?

e) What about other ways, aside to conversations, you may have conveyed information about alcohol and ways to stay safe?

- *Providing them with social media stories?*
- *Suggesting online sites to get further age-appropriate information?*
- *Texting or non-verbal tips over staying safe?*
- *Banned alcohol within the home?*
- *Disciplined them over an alcohol-related event?*

1. **Ease and difficulties in holding conversations - facilitators and barriers *(chance to reflect on their discussion in the interview)***

a) In general, how easy or difficult have you found these conversations about alcohol?

- *What things are easy to talk about and what things are difficult to talk about?*
- *What types of questions or topics do you find easy or more difficult to answer?*
- *Do you feel confident in your own knowledge about talking to your child about alcohol? (understanding of legal issues, units, risks of alcohol use) – areas more/less confident in?*
- *Have you felt there are any particular barriers that have prevented you from talking to your child about alcohol?*
- *Would you like to talk to your child more about alcohol than you do?*

b) Are there any topics that you feel are not appropriate at this age – suitable for when they were younger or when they are older?

1. **How do you think we can help you and other parents have these important conversations about alcohol? *(moving from own experiences to what could help them and other parents in the future to have effective conversations about alcohol)***

a) Do you think you have enough support/information to talk about alcohol with your child?

*Probe. If yes, ask them to expand on this.*

*If no, would you like more support/information about communicating about alcohol with your child? What type of support/information would you like?*

b) Have you used any resources (such as internet, leaflets etc.) to help you talk about and supervise alcohol in the family?

c) Are there any particular topics that you’d like more support with in talking with your child?

*What topics would you like to see covered in any materials e.g. legal issues, health issues, safety issues, why children drink, how to prevent your child drinking alcohol.*

*Think about any issues that you your child particularly needs/wants about alcohol?*

d) What format would you prefer to have this information/support materials?

*Hard copies such as leaflets, online information, telephone app?*

e) In general, how do you think we can help parents talk more to their children about alcohol?
