# Supplementary material for: Understanding conversations about alcohol between parents and their 15–17 year olds: a qualitative study
Source: BMC Public Health. 2018 May 16;18:631. doi: 10.1186/s12889-018-5525-3 (PMC5956849; doi:10.1186/s12889-018-5525-3)
Supplement: Supplementary file 2 — Topic guide – young people (DOCX 26 kb) [file 12889_2018_5525_MOESM2_ESM.docx]

**TOPIC GUIDE – INTERVIEWS WITH YOUNG PEOPLE**

1. **Introduction**

a) Before we start properly, can I just ask a few questions about yourself to provide a bit of context to the rest of our discussion? (age, education, ethnicity)

1. **Alcohol in the home - parent and child’s use** *(introduce the topic of alcohol use in general terms and explore attitudes including any principles they have around alcohol whether personal or institutional including religion)*
2. As far as you know do your parents drink at home?

*If yes, ask them approximately, how often? In front of you/children?*

*In general, do you think that parents’ drinking has an effect on young people?*

*Do you have any views about alcohol in the home, such as its visibility or consumption? This could be a personal viewpoint or an institutional one from religion or wider belief system.*

*If no, do you think parents’ drinking style may have an effect on young people?*

*Do you have any views about alcohol in the home, such as its visibility or consumption? This could be a personal viewpoint or an institutional one from religion or wider belief system.*

b) Have you ever drunk alcohol?

*If yes,* *roughly how much do you drink, what do you drink, how often do you drink, who do you drink with, and where do you drink? Do you like drinking and the effects it provides? When did you have your first whole drink of alcohol (not a sip)? Who were you with when you first had this first drink?*

*Do your parents know if/how much you are drinking/who with/what happens when you drink?*

*Do your parents try to limit (or influence) your drinking in any way, for example, by cutting down the amount, or how often you drink, or who you drink with, or where you drink, type of drink, staying safe whilst drinking, etc.*

*If no,* *are there any reasons why you have not tried alcohol? This could be a personal viewpoint or an institutional one from religion or wider belief system. Could be taste, effects, conflict with other activities like sport?*

1. Have your parents tried to monitor/supervise alcohol in the family?

*If yes, how have they have done this? Do you think it has been successful? What do you think of the ways your parents have tried to do this?*

1. Where do you get information about alcohol from*?*

*Probe. Social media, friends, school, internet, TV, magazines.*

*What do you think about this information and the situation when it was provided?*

1. How would you judge your knowledge about alcohol?
2. Do you have any concerns about drinking alcohol?

*If yes, what worries you the most? Probe. Health issues (being sick, falling over, getting into fights, damage to body); practical issues (where they drink, type of alcohol, availability to young people, amount they drink), drinking culture, being vulnerable dangers that young people may be exposed to through drinking), regretted events including social media contributions*

1. **Talking to your parents about alcohol with view of importance and examples**
2. In general, do you think it is important to talk about alcohol with your parents?

*Do you think it is your parents’ role to talk to you about alcohol? (compared to school, other sources, etc.)*

1. Have your parents ever spoken to you about alcohol? For example, about its effects, how to stay safe, legal issues?

- *Ask children to expand on the details of the conversations that they have had with their parents – to include how it was brought up and information/topics conveyed;*
- *How did you find this conversation?*
- *Can you remember when your parents first spoke to you about alcohol? (separate out pre 15 and post 15- year old conversations)?*
- *Did your parents use or refer to any social media (websites etc.) when talking to you about alcohol?*

c) If your parents haven’t spoken to you about alcohol why do you think this is?

*Is it important to you that your parents speak to you about alcohol or not really? Have you wanted to speak to your parents about alcohol but couldn’t?* *Would you prefer your parents to bring up the conversation about alcohol, rather than yourself?*

1. In general do you think you know more about drinking than your parents do? *For example, things may have changed from when they were young.*
2. **Talking in more detail - Raising the issue *(moving into further detail about conversations starting with raising the issue)***

a) How did the topic first come up? Did you or your parents raise the issue?

- *Easier to respond to their questions or easier to start the discussion with them?*

b) When do you think are the best times for your parents to talk to you about alcohol?

*Probe. Opportune moments, at dinner, night-time, when getting a lift somewhere (car journey), when something has triggered the need to talk about alcohol, when you show interest/curiosity – when would be the worst times?*

1. When speaking to your parents about alcohol, what do you think have been the most useful ways they have started a conversation? *Probe.* *Using real-life examples (personal, family, or peer experiences), using your language, using resources and digital media, etc*

- *What do you think would be the most useful ways for parents to start a conversation about alcohol?*

1. **Talking in more detail – Topics *(moving from how to start conversations to information/topics shared)***

a) What information about alcohol would you like your parents to discuss with you?

*Probe. Information about alcohol; legal issues; safety issues [Identify where discussion of* *alcohol-harm reduction occurs in these conversations]; social media – regretted posts, used to keep safe (‘checking in’); sex and relationships rules and expectations about alcohol use, how to cope with peer pressure.*

1. **Examples of conversations (*to focus on specific examples if this hasn’t arisen already in the interview to capture the precise details of what was said, when etc.)***

a) I’d like you to think about some instances where you’ve had a conversation with your parents about alcohol – think about one example where you felt it went well (like they got some useful information across, raised at a good time etc.)

b) And now think about a situation where it didn’t go so well – why was that?

1. **Most/least effective conversations *(strong focus on a particularly effective conversation and other strategies so that such real-life tips and techniques can be shared with other parents)***
2. Have your parents ever spoken to you about the best ways to reduce harm when drinking?

*Probe. Eating properly, plans to return home safely, drinking in groups and ‘looking out for each other’, nominated lower level drinker, watching drinks for fear of spiking, social media to ‘check in’ with friends/family.*

1. Overall, what do you think have been the best conversations your parents have had with you about reducing current/future use or harm from alcohol?
   - *Believing facts parents offer about alcohol*
   - *Understanding and agreeing with parents’ concerns*
   - *Agreeing rules around alcohol*
   - *Strategies ant tips*
2. **Reflecting on your conversations with your parents *(chance to reflect on their discussion in the interview)***

a) In general, how have you felt/do you feel about talking to your parents about alcohol?

*Probe. comfortable, uncomfortable, challenging, straightforward*

- *What things are easy to talk about and what things are difficult to talk about?*
- *What makes it easy or difficult to talk about alcohol with your parents*?
- *Does anything worry you about having conversations with your parents about alcohol?*

1. Generally, do you follow the advice that your parents give you about alcohol?
2. Would you like to talk to your parents more about alcohol than you do?

*Do you feel happy with the level of communication? If not, why not?*

1. **How do you think we can help parents have these important conversations about alcohol with their children? *(moving from own experiences to what could help them and other parents/children in the future to have effective conversations about alcohol)***
2. In general, how do you think we can help parents, in general, talk more to their children about alcohol?
3. Who do you think the best person is to talk to you about alcohol? Why is this?

*Probe. if not the parent explore what role the parent could play when it comes to provision of information and guidance on alcohol.*

1. What might you say to your children or younger brothers and sisters (or other young people) about alcohol? What advice might you given them? What advice do you wish that you had been given when you were younger?
2. What do you think makes young people most likely to remember/follow advice or rules at the point when they’re being offered alcohol – if anything?
